# Marginalizing the Mainstream: How Social Media Privilege Political Information

**DOI:** 10.3389/fdata.2021.689036

**Published:** 2021-07-06

**Authors:** Richard Rogers

**Affiliations:** Media Studies, University of Amsterdam, Amsterdam, Netherlands

**Keywords:** misinformation, fake news, social media, data journalism, web epistemology

## Abstract

The following reports on research undertaken concerning the “misinformation problem” on social media during the run-up to the U.S. presidential elections in 2020. Employing techniques borrowed from data journalism, it develops a form of cross-platform analysis that is attuned to both commensurability as well as platform specificity. It analyses the most engaged-with or top-ranked political content on seven online platforms: TikTok, 4chan, Reddit, Twitter, Facebook, Instagram and Google Web Search. Discussing the extent to which social media platforms marginalize mainstream media and mainstream the fringe, the analyses found that TikTok parodies mainstream media, 4chan and Reddit dismiss it and direct users to alternative influencer networks and extreme YouTube content. Twitter prefers the hyperpartisan over it. Facebook’s “fake news” problem also concerns declining amounts of mainstream media referenced. Instagram has influencers (rather than, say, experts) dominating user engagement. By comparison, Google Web Search buoys the liberal mainstream (and sinks conservative sites), but generally gives special interest sources, as they were termed in the study, the privilege to provide information rather than official sources. The piece concludes with a discussion of source and “platform criticism”, concerning how online platforms are seeking to filter the content that is posted or found there through increasing editorial intervention. These “editorial epistemologies”, applied especially around COVID-19 keywords, are part of an expansion of so-called content moderation to what I call “serious queries”, or keywords that return official information. Other epistemological strategies for editorially moderating the misinformation problem are also treated.

## Introduction: The Politics of Problematic Information and Its Cross-Platform Study

While scholars of hearsay, rumor and conspiracism would point to the history of its staying power (Olmsted, 2009), the spread of misinformation and other problematic information is said to be “supercharged” by contemporary social media (Bounegru et al., 2018; Daniels, 2018). The following examines that thesis through an analysis of the current state of what globally could be called the “misinformation problem” (Allcott et al., 2019) across seven online platforms: TikTok, 4chan, Reddit, Twitter, Facebook, Instagram, and Google Web Search. The part played by YouTube is viewed by way of the videos referenced on 4chan. The case in question is the political information environment in the run-up to the U.S. presidential elections, or what may be dubbed U.S.-based, “political Facebook”, “political Twitter”, “political Instagram”, etc. Borrowing a technique from data journalism, and examining the most interacted-with content around the candidates, political parties and election-related issues, the work reported here found that stricter definitions of misinformation (imposter sites, pseudo-science, conspiracy, extremism only) lessen the scale of the problem, while roomier ones (adding “hyperpartisan” and “junk” sites serving clickbait) increase it, albeit rarely to the point where it outperforms non-problematic (or more colloquially, mainstream) media.

The misinformation problem differs per platform. On such youthful platforms as TikTok and to a lesser extent Instagram, misinformation may be delivered sarcastically or insincerely, making it difficult to characterize intent (Phillips and Milner, 2017). On the masked or anonymized political boards and communities of 4chan and Reddit, problematic sources are not as copiously referenced as mainstream ones, but that finding does not mean to suggest the absence of a problem, as the most referenced collection of sources are extreme YouTube videos, many of which end up being deleted from the platform. The users of mainstream social media as Twitter and Facebook continue to point in great proportions to hyperpartisan sources, originally defined as “openly ideological web operations” (Herrman, 2016). Political spaces on Instagram, however, were found to be the “cleanest”, where most election-related content is non-divisive and earnestly posted, and influencers, with some exceptions, were found to be responsible information providers, debunking rather than spreading 5G coronavirus conspiracy theories.

The research provides a technique for “cross-platform analysis”, or the examination of a single phenomenon (through engagement analysis) across a variety of social media. It thereby addresses critiques of “single platform studies”, where societal trends or phenomena are seen through one social media lens without the benefit of a comparative perspective that would furnish a baseline (Rogers, 2019). Engagement analysis of a single subject matter (election-related information, in this case) is considered one robust cross-platform approach since it captures each platform’s top content, which refers to the posts or web URLs that receive the most interactions (directly or indirectly). It has the benefit of being more global in its outlook compared to other cross-platform approaches that rely on seeking one or more digital objects shared across platforms (e.g., a hyperlink) and comparing resonance (Rogers, 2017).

But the cross-platform approach put forward here is not blind to platform specificities. It seeks to account for differing platform metrics, vernaculars of use and user subcultures. Accounting for this “medium specificity” is performed in at least three ways. The first is that engagement is measured distinctively per platform, as discussed in some detail below. Second, social media manipulation (such as artificially amplifying misinformation so that it appears to be engaged-with content) also differs per platform. One is interested in fake followers on Instagram and bots on Twitter, for example. Being attuned to platform vernaculars, finally, rests on the study of cultures of use. For example, certain sound effects or facial gestures on TikTok suggest disbelief or mistrust. In all, commensurability thereby relies on both the cross-platform study of engagement as well as individual platform analyses imbued with a medium-specific approach.

In the following I first introduce the current misinformation problem online as bearing some resemblance to the quality of information debates from early web history. The contemporary concerns, however, flow from the “fake news” crisis of 2016, together with the continual study of the extent to which the platforms have addressed the issue (and also how they have done so). Moreover, these debates have not escaped the politicization of “big tech” and its supposed “liberal bias” (Vaidhyanathan, 2019), a claim that is also a source of empirical study in the Google Web Search analysis below.

Indeed, designating certain information as problematic may be political (and increasingly politicised), because, as others before us also have found (Benkler et al., 2018; Rogers and Hagen, 2020), it is more prevalent on the right side of the political spectrum, as are problematic or “inauthentic” users, though they are not alone there. Making a case for balancing the partisanship of sources outputted by social media and search engines (rather than serving filter bubbles through personalization, for example) is among the emerging source adjudication methods under consideration, as I will discuss. The piece concludes with a discussion of source criticism on social media, including the recent rise of “editorial epistemologies” alongside crowdsourced ones associated with the (early) web.

## Uncertainty Online Renewed

The web historically has been thought of as a space for the unsubstantiated, authored by rumour-mongers, conspiracy theorists and all manner of self-publishers and fringe contributors. Indeed, one could argue, as it was put in 1994, that on the web the “eminent and the crackpot” stand side-by-side, a feature once celebrated as a productive collision (Rheingold, 1994; Rogers, 2004). Indeed, in early internet studies, next to the blurring of the real and the virtual, conspiracy theory in particular but also the production and circulation of rumor were subjects of study, before notions as the “wisdom of the crowd” and projects as Wikipedia appeared to place the web on a less shaky epistemological footing (Dean, 1998; Shirky, 2008). Arguably, social media have put paid to that brief period of relative stability. Conspiracists or at least those who discuss such phenomena as the link between 5G and the coronavirus are among some of the high-profile influencers or microcelebrities found there (Bruns et al., 2020). In turn, scholars now write, as they did two decades earlier, that the internet is “mainstreaming the fringe” (Barkun, 2016).

The recent uptick in attention to the study of problematic content online could be attributed as well to the “fake news crisis” of 2016, where it was found that so-called fake news outperformed mainstream news on Facebook in the run-up to the U.S. presidential elections that year (Silverman, 2016). That finding also set in motion the subsequent struggle around the occupation of the term from a type of news originating from imposter media organisations or other dubious sources to a “populist” charge against mainstream and “elite” media that seeks to delegitimate sources found publishing inconvenient or displeasing stories (van der Linden et al., 2020). In its recent study we have had calls to cease using the term, fake news (Pepp et al., 2019). There also has been a series of classification moves. Both the expansion as well as contraction of the notion may be seen in its reconceptualization by scholars as well as by the platforms themselves (Venturini, 2019). The definitional evolution is embodied in such phrasings as “junk news” and “problematic information”, which are broader in their classification, while the platforms appear to prefer terms such as “false” (Facebook), which is narrower (Rogers, 2020a). On the back-end the platform companies also develop responses to these activities. They would like to automate as well as outsource its detection and policing, be it through low-wage, outsourced content moderators (volunteer) fact-checking outfits or user-centred collaborative filtering such as Twitter’s “birdwatchers”, an initiative they say born of societal distaste for a central decision-making authority, as found through qualitative interviews (Gillespie, 2018; Roberts, 2019; Coleman, 2021). They also take major decisions to label content by world leaders (and indeed have world leader content policies), which subsequently land platform governance and decision-making in the spotlight (Twitter, 2019).

More broadly there has been a rise in the study of “computational propaganda” and “artificial amplification” which the platforms refer to as “inauthentic behavior” (Woolley and Howard, 2016; Colombo and De Gaetano, 2020). These may take the form of bots or trolls; they may be “coordinated” by “troll armies”, which has been outlined in Facebook’s regular “coordinated inauthentic behavior reports” (Facebook, 2021). As its head of security policy puts it, Facebook defines it (in a plain speak manner) as “people or pages working together to mislead others about who they are or what they are doing” (Facebook, 2018). Occasionally data sets become available (by Twitter or other researchers) that purport to be collections of tweets by these inauthentic, coordinated campaigners, whereupon scholars (among other efforts) seek to make sense of which signals can be employed to detect them (Roeder, 2018).

Other types of individuals online have caught the attention of the platforms as “dangerous” (Facebook), and have been deplatformed, a somewhat drastic step that follows (repeated) violations of platform rules and presumably temporary suspensions (Rogers, 2020b). “Demonetisation” also is among the platforms’ repertoire of actions, should these individuals, such as extreme internet celebrities, be turning vitriol into revenue, though there is also the question of which advertisers attach themselves (knowingly or not) to such content (Wilkinson and Berry, 2020). Moreover, there are questions about why certain channels have been demonetized for being “extremist”. Others ask, is “counter-speech” an alternative to counter-action (Bartlett and Krasodomski-Jones, 2015; Gagliardone, 2019)?

On the interface, where the metrics are concerned, there may be follower factories behind high follower and like counts (Lindquist, 2018). The marketing industry dedicated to social listening as well as computational researchers have arrived at a series of rules of thumb as well as signal processing that aid in the flagging or detection of the inauthentic. Just as sudden rises in follower counts might indicate bought followers, a sudden decline suggests a platform “purge” of them (Confessore et al., 2018). Perhaps more expensive followers gradually populate an account, making it appear natural. Indeed, there is the question of which kinds of (purchased) followers are “good enough” to count and be counted. What is the minimum amount of grooming? Can it be automated, or is there always some human touch? Finally, there is a hierarchy in the industry, where Instagram followers are the most sought after, but “influencers” (who market wares there) are often contractually bound to promise that they have not “participated in comment pods (group “liking” pacts), botting (automated interactions), or purchasing fake followers” (Ellis, 2019).

Having touched upon the current state of uncertainty online, I would like to turn to how problematic information manifests itself in social media platforms around specific issues. The following recounts a cross-platform analysis into the “misinformation problem” in the run-up to the 2020 U.S. presidential election. As noted above, the overall approach is to study most engaged-with content with a sensitivity to platform metrics, vernaculars of use and user subcultures. It relates a set of empirical studies that enquire into the extent to which platforms are again mainstreaming the fringe, examining more specifically those spaces conjured through “serious queries” that contain election-related as well as COVID-19 information (Rogers, 2021). When querying political hashtags, candidate and party names as well as issues, and sifting through the content most interacted with on the platforms, how do more mainstream sources fare in comparison to those characterized as problematic? More to the point, is social media marginalizing the mainstream?

Here I take the most salient findings per platform in turn, before concluding with a discussion of the emergence of editorial epistemologies put into use by social media platforms as well as search engines. Editorial source adjudication is a remarkable transformation in how these platforms sift and filter sources, indicating an exceptional information state, a point upon which I conclude.

## TikTok: Instilling Doubt in Mainstream Accounts

TikTok is not usually considered a site for political encounter, but recently the short video sharing platform, used predominantly by youth, has posted rules about political content, indicating its growing presence there. It also warns against “misleading information” and urges users to “verify facts using trusted sources”, suggesting that misinformation could be worthy of investigation on the platform (Mahendran and Alsherif, 2020). Apart from how to locate political content, we asked, how do TikTok users express themselves politically (Sánchez-Querubín et al., 2021)? How may forms of creative expression on TikTok manifest themselves as misinformation?

TikTokers employ a range of creative expression such as singing, dancing, duet, lip-syncing, mimicking, finger dancing, viral sounds and facial expressions. Some have specific connotations for TikTok insiders and make for trends. Having queried election related hashtags, such as #trump2020 and #biden 2020, it was found first that TikTokers make copious use of political hashtags, attaching both Trump and Biden-related hashtags to the same video, thereby striving to maximize the audience and view counts, rather than identify with one candidate or another. In the analysis, the researchers undertook a format and content analysis of the top 30 videos per hashtag query, examining which forms of creative expression are used in political videos and where misinformation may be imparted.

Three forms stood out: lip-synching, viral sounds and facial expressions. TikTokers match the lip movements of the candidates, often sarcastically, for example when the comedian, Sarah Cooper, 2020 lip-synched Trump’s remarks during a White House briefing on using ultraviolet light and detergent to thwart the coronavirus (2020). The other forms of creative expression of interest here are viral audio and facial expression. For instance, “Ride It” by Regard is a viral sound that is often paired with finger dancing to relate stories of cultural misunderstanding. It was found that TikTokers use it when dealing with accusations of being a Trump supporter, such as “get called racist 24/7” (sound), “get yelled at for presenting facts” (sound), and “accused of not respecting women” (sound). The viral sound denotes being misunderstood, eliciting sympathy but also a knowing smile.

Finally, the facial expression that approximates the doubtful emoji is another creative expression often encountered. In these videos news footage may be cut into the shots, such as multiple clips of Joseph Biden hugging women, with the intention to sew doubt about his fitness for presidential office. Here we found many of the political videos instilling mistrust in news clips through sarcastic and doubtful facial expressions. Such a finding prompted consideration of adding “instilling mistrust” as a category to the spectrum of misinformation types developed by Wardle (2017), which ranges from parody (least intent to deceive) through misleading content and false context to fabricated (most intent). Alternatively, one could argue that on TikTok all categories of misinformation could be hybridized, for TikTokers are employing parody when simultaneously introducing misleading content, false context or other misinformation types.

## 4chan and Reddit: Referencing Extreme YouTube Content

Unlike public-facing platforms such as Facebook and Twitter, where users cultivate an online self, 4chan and Reddit are so-called masked spaces of anonymous users (De Zeeuw and Tuters, 2020). Particularly the board, 4chan/pol, and the subreddit, r/TheDonald, have been associated with election politics, and especially the 2016 Trump campaign, where support for his candidacy took the form of “the great meme war”, which comprised the deployment of vernacular language, image macros and other tactical media to support the candidate’s cause (Donovan, 2019). Previous research into misinformation in 4chan/pol and across Reddit found little reference to outwardly problematic sources, such as imposter news sites or (Russian) disinformation, but rather numerous links to extreme videos on YouTube that were later removed (Hagen and Jokubauskaitė, 2020). Thus, while not necessarily a space that links to disinformation sources, it is problematic for other reasons.

Here, in the context of the run-up to the U.S. presidential elections of 2020, the research enquired into the extent to which U.S.-based political boards and forums on 4chan and Reddit share misinformation and “junk” content, and more specifically imposter news and other types of “pink slime” websites, termed as such for the use of low-cost, newspaper-like sites often publishing repurposed content (Tarkov, 2012; Bengani, 2019). We also were questioning the interest these boards and communities might have in what has been termed an “alternative influence network”, a group of extreme social media influencers that “facilitates radicalization” (Lewis, 2018). The research employed the so-called “needle-to-haystack” technique, querying 4chan/pol and all of Reddit for the URLs of the pink slime websites, and the “haystack-to-needle” technique which queries an expert list of problematic sources (hosts) in the same platform datasets (Hagen and Jokubauskaitė, 2020).

No pink slime sites were encountered, suggesting either their lack of significance (despite returning high in Google queries) or the media literacy of the users on the boards and communities (or both). Scant amounts of problematic sources were found but like in previous research copious YouTube links were identified, which led to the inquiry into whether YouTubers from the alternative influence network are significantly present in those online cultures. The alternative influence network is described here as a set of YouTube channels fluctuating between “news and personality-centric vlogging, spreading misinformation-laden commentary” (Burton and Koehorst, 2020). Indeed, many of these channels were found between the boards and subreddits under study, though their presence was unequally distributed. 4chan/pol and Reddit are rather different in their media consumption, with political Reddit preferring to reference videos using the “alternative debate style” and/pol electing more for the “toxic vox populist” style of single person, direct-to-audience (Tuters and Burton, 2021). Indicating their extreme speech, a significant percentage of the YouTube videos referenced on 4chan has been removed by the platform.

## Twitter: Hyperpartisan Sources in Ascendancy

Like Facebook and to a lesser extent Instagram, Twitter also has been the focus of public attention concerning misinformation around the 2016 United States presidential elections and beyond. Twitter, rather unlike the other two platforms, has aided researchers in its study through providing curated data sets of Russian and alleged Iranian trolls and influence campaigners, or what are referred to as inauthentic users (Gadde and Roth, 2018). Thus, in the study of misinformation on Twitter, there are generally two strands of analysis to consider—problematic content as well as users. During the 2016 election campaigning and running through to at least late 2019, much of that content and those users, described as “sprawling inauthentic operation [s]” were promoting “pro-Trump messages” (Romm and Stanley-Becker, 2019).

Here we revisit these claims through a study of the content and users on “political Twitter” in the early run up to the 2020 United States presidential elections, where we examine result sets of queries for election-related hashtags and keywords, together with the users most active in deploying them (Groen and Geboers, 2021). How much problematic information is present in the most interacted-with content on political Twitter? Are problematic users among the most active? Are they generally of a particular political persuasion?

The content under study are the URLs (hosts) that are referenced in the tweets, and the most active users defined as those who tweet the most. In a three-week timeframe prior to and just after Super Tuesday (March 2020), when a cluster of election primaries and caucuses were held, we compared the hosts to a list of problematic sources curated by combining pre-existing labeling sites, including (Allsides, 2020; Media Bias/Fact Check, 2020; Otero, 2020) “the Chart”, and NewsGuard, 2020. We also consulted Wikipedia and other news sources mentioning the sources in question. With one exception (related to the query DACA, the immigration issue), we found little reference to disinformation, imposter news sources, pseudo-science, conspiracy theory sources or extreme sites. When expanding the definition of problematic information to include hyperpartisan sites, however, over half would fall into that category, with the implication that social media (or at least a goodly share of users of “political Twitter”) appear to marginalize mainstream sources. Put differently, if we were to employ Craig Silverman’s original definition of “fake news”, it could be said to outperform mainstream sources anew, as it had in the immediate run-up to the 2016 United States elections (on Facebook) (2016).

For the study of the most active users, we analyzed “authenticity” with the aid of Sparktoro, 2021 which employs indicators (abnormal tweeting activity, unusual combinations of followers/following, etc.) to make a determination. We also studied user partisanship or side-taking through qualitative profile analysis. Our findings are not dissimilar to others in that there is far more inauthenticity in the pro-Trump user base, but we also found that there are a few flagged users on the other side of the political spectrum, too.

## Facebook: The Seminal “Fake News” Problem Persists

Journalists began calling Facebook the seminal “fake news machine” (Gallucci, 2016) just after the finding made by *Buzzfeed News* that so-called fake news was liked and shared more than mainstream news on the social media platform in the three months prior to 2016 U.S. presidential elections (Silverman, 2016). Since then, there has been a steady stream of stories from Facebook’s corporate blog concerning both its crackdowns on “inauthentic coordinated behavior”, or influence campaigning, as well as its initiatives to curb misinformation and “false news”, which is a narrow definition including pseudo-science and conspiracy sites though excluding hyperpartisan ones (Mosseri, 2017). The measures began in at least april 2017 with among other plans to economically disincentivize such sources as the infamous Macedonian fake news factory that chose divisive pro-Trump messaging (over pro-Sanders’) because it brought in far more revenue (Silverman and Alexander, 2016; Tynan, 2016). Has much has changed in how well “fake news” is consumed on the platform since 2016?

Researchers and I revisited the original *Buzzfeed News* story and its data journalism method in order to investigate the state of the “fake news” problem in Jan-March 2020 (Rogers, 2020), which is roughly the first of the three timeframes under consideration in the original *Buzzfeed News* piece entitled, “viral fake election news stories outperformed real news on Facebook” (Silverman, 2016). Though it would change dramatically, in Feb-Apr 2016, the share and like counts of mainstream news were well ahead of those of “fake news”, which Silverman defined as sources ranging from “hoax sites [to] hyperpartisan blogs”. Using election-related queries in BuzzSumo, 2020, the social media research and monitoring tool, we compiled a list of sources and characterized them with the aid of a series pre-existing “bias” labeling sites (including (AllSides, 2020; Media Bias/Fact Check, 2020; Otero, 2020) “the Chart” and NewsGuard) so that we had a rough indication of their quality and partisanship. Sources are categorized as problematic or non-problematic (which more colloquially could be called “mainstream”), and those falling into the latter category were subcategorized as (hyper)partisan-conservative (hyper)partisan-progressive or neither of the two, again with the aid of the existing labeling sites. Problematic sources included imposter news (and so-called “pink slime” sites), pseudo-science, conspiracy theory and extreme sites, as was done in the Twitter study above (Bengani, 2019).

If we continue to use Silverman’s “fake news” definition (that includes hyperpartisan sites) then Facebook’s fake news problem has worsened slightly. Compared to the same period in 2016 the proportion of “fake news” engagement to that of mainstream news has increased from roughly 1 in 4 to 1 in 3.5. If, however, we tighten the definition, as Facebook has done, to “false news” and include in that category only the sources or stories flagged as “problematic” the scale of the problem drops substantially to 1 in 9. It should be noted that we encountered no imposter news sites, which may suggest that they are well targeted by Facebook or that they are not significantly resonating among users.

Nonetheless, in the last period under study in 2016, when Silverman found that “fake news” performed well, imposter sites (as the Denver Guardian) comprised a majority of those most interacted-with. One implication of the finding is that efforts to identify imposter sites (and other “pink slime”) continue to have value, despite the fact that they are not yet well consumed. Another implication is that if the problem remains of a smaller scale, scaled-up fact-checking may continue to find its place among the counter-initiatives, rather than only mass content moderation and automation.

## Instagram: Influencers as Responsible Information Sources?

Instagram had been one of the more understudied and under-appreciated social media platforms when it came to misinformation. That changed with the release of two major reports on the Russian disinformation campaigning surrounding the 2016 United States presidential elections (DiResta et al., 2018; Howard et al., 2018). In fact, in one study, it was noted that unlike the other social media platforms Instagram actually saw a rise in disinformation activity in the period just after the elections (Howard et al., 2018). Many of the posts, including memes, were openly divisive, but others were sarcastic and more difficult to decode with respect to stance or side-taking. As scholars have found, over the past few years more and more content online could be described as equivocal or ambivalent, where the sincerity of the post and the sender is unclear (Phillips and Milner, 2017; Hedrick et al., 2018).

In the study of election-related Instagram posts in the early run-up to the 2020 United States presidential elections (Jan-April 2020), we enquired into the amount of divisive and ambivalent posts, compared to non-divisive and earnest ones (Colombo and Niederer, 2021). How sarcastic and “edgy” are the top election-related posts on Instagram? Does it form the dominant mode of political discourse on the platform? We also are interested in whether misinformation is spread in this divisive, ambivalent style. To begin to answer these questions, we queried CrowdTangle, Facebook’s content monitoring tool, for the names of the candidates and select social issues (healthcare, gun control, COVID-19 and 5G), and coded the top 50 posts for divisiveness (or non-divisiveness) and ambivalence (or earnestness), whereby each post is ultimately given a hybrid label, e.g., divisive-ambivalent. We scrutinized the candidate-or issue-related posts by influencers that had particularly high engagement scores, often at the top of the rankings. We also sought misinformation.

Perhaps counter-intuitively, we found that the vast majority of the top posts concerning the candidates as well as the social issues are earnest and non-divisive. Virtually no posts were found to be divisive and ambivalent. Indeed, most posts were sincere expressions of support. Of the few divisive posts which the coders additionally found to be earnest, half were by Donald Trump or Donald Trump, Jr, and most of the rest concern Trump or gun control. Apart from a few posts pushing a conspiracy theory surrounding 5G and COVID-19 (including one post that ranked second in engagement), no other misinformation was encountered. The top 5G related post, by an influencer, debunked the conspiracy. Indeed, with a few exceptions we also found that the influencers were posting responsibly and earnestly.

In a separate exercise we studied the authenticity of the followers as well as the political parties, employing the HypeAuditor, 2020 tool. While the Republican Party’s account had over 25% of suspect followers, and Trump’s had 25%, the other candidates and party were not far behind at 20%. It should be noted, though, that when separating the two categories that make up inauthentic followers—“mass follower” accounts and “suspect” accounts—the Republican Party and Trump tally higher on suspicious followers, defined as “Instagram bots and people who use specific services for likes, comments and followers purchase” (Komok, 2018).

## Google Web Search: Liberal Sources Outnumber Conservative Ones

While Google Web Search could be considered the dominant information machine online, among the major platforms and online services it has been one of the least studied for misinformation. Recognizing the potential for its spread during the pandemic, or what the head of the WHO called the “infodemic” (UN DGC, 2020), Google has been curating the results for queries concerning the COVID-19 pandemic, with side bars ordering the official information served, and results geo-tailored to provide local and national resources. Such information curation is considered unprecedented, unless one counts Google’s disclaimer notice on top of the results page for the query “Jew” (Sullivan, 2004), or the cleaning up of autosuggested queries to remove ethnic, homosexual and other slurs (Gibbs, 2016). Another contemporary context behind the study of election-related Google results concerns the debate surrounding “liberal tech bias” (Schwartz, 2018). Could Google results be thought to exhibit a bias toward or against particular types of sites? How to characterize the sites returned for political queries?

In order to start to answer these questions, we queried candidate names, political parties and a host of election-related issues in Google, with results from the “United States region” from January 12, 2019 to March 23, 2020 (Torres, 2021). In an examination of the top 20 results per query, we ask, how to characterize the sources returned? Are problematic sources present and even highly ranked? How could the results be characterized politically? To do the analysis, we curated a source list of problematic and non-problematic sources, largely news and cultural commentary, combining a set of media labeling sources, as in the Twitter and Facebook projects discussed above. We also consulted Wikipedia and online news mentions of potentially problematic sources. The categorization is considered rough and is meant to give an indication rather than a determination. With the aid of the labeling sites, we also assigned political leanings. There are two distinctive political categorization schemes, one “ample” and one “narrow”, with the former merging center-left and left and center-right and right, and the latter only including explicitly left (liberal) or right (conservative) labels, according to the sites that sort sources in such a fashion. (When there was disagreement among the labeling sites, we went with the majority.) We also labeled the sites returned that fell outside the categories, such as “special interest”, “local news” and “official”.

In all we found that the Google results for our nearly 120 queries resulted in scant problematic information returned. Hardly present as well were official sources that we defined as federal or local government, intergovernmental agencies, politicians, or campaign websites. Special interest sites, a broad category ranging from think tanks to advocacy groups, have an outsized presence in the results, however. These sites tend to specialize in an issue or industry, which is also an indication of how Google values information sources. Most significantly, when considering the political leanings of sources, it is striking that Google could be said not to seek “balance”. That is, liberal sources outnumber conservative ones in the results for all queries made. Employing the “ample” categorization, the results were 6:1 in favor of liberal sources, and 3:1 when employing the narrower scheme.

## Mainstreaming the Fringe And/Or Marginalizing the Mainstream?

At the outset the question to be addressed concerned the extent to which social media is “mainstreaming the fringe”, not so unlike the early web, prior to the development of epistemologies that placed it on firmer ground. Among those mentioned were the wisdom of the crowd such as Wikipedia’s collaborative editing, but there were others. For instance, Yahoo! and DMOZ employed librarianship in their directory-making, Google used hyperlink analysis scientometrically, and the early United States blogosphere constituted a kind of fact-checking, epistemic community, most famously uncovering faked documents held up as authentic by an authoritative TV news program (60 Minutes), in what has become known as the “Killian documents controversy” (Callery and Proulx, 1997; Langville and Meyer, 2006; Wikipedia contributors, 2020). Here we now ask the same of social media. How to characterize the current epistemological foundations of online platforms?

In order to grapple with that question, I briefly sum up the findings with respect to the relationship between the mainstream and the fringe per platform and draw conclusions from our cross-platform approach. Generally speaking, social media and its users appear to be marginalizing the mainstream. Subsequently, I discuss the prospects of source adjudication in terms of results curation or otherwise managing which content is allowed to remain on social media platforms. It is a form of “platform criticism” that speaks to the various emerging epistemologies on offer to stabilize social media.

The social media platforms under study have varied relationships with mainstream media, at least with respect to those sources or posts most interacted with in the early run-up to 2020 United States presidential elections. Broadly speaking, TikTok parodies it, 4chan and Reddit dismiss it and direct users to alternative influencer networks and extreme YouTube content. Twitter prefers the hyperpartisan over it. Facebook’s “fake news” problem also concerns declining amounts of mainstream media referenced. Instagram has influencers (rather than, say, experts) dominating user engagement. By comparison, Google Web Search buoys the liberal mainstream (and sinks conservative sites), but generally gives special interest sources, as they were termed in the study, the privilege to provide information rather than official sources.

Given the decline of what one could call “mainstream authority” online, how to characterize the contemporary approaches to source adjudication, when considering problematic information? That platforms are manually editing results (for certain queries) indicates what I would call an “exceptional information state”.

Recently, social media platforms and Google web search have begun to curate the results of such “serious queries” as coronavirus, COVID-19 and similar terms related to the global pandemic. Such filtering may explain the scant amount of outwardly problematic information such as conspiracy websites encountered in the top results for coronavirus queries across the platforms. It does, however, raise the question of the epistemology behind the authority that is being applied, and whether it puts paid (for example) to the signals approach of algorithms, and instead puts forward “editing in” official sources as the top content recommended.

## Editorial Epistemologies

Source list or results curation is laborious work and fell into decline with the overall demise of the human editing of the web and the rise of the back-end, and algorithmic signals, taking over from the editors (Rogers, 2013). COVID-19 and the coronavirus are thus exceptional for they have marked the return of the editors and raise the question of whether their work should extend beyond pandemic sources to election-related information, as discussed above. Maintaining COVID-19 and the coronavirus as an exceptional information state would draw the line there, though cases could be made to extend the adjudicative practice to the democratic process, where policymakers especially in Europe have directed their efforts. France’s false news legislation comes to mind, as does Germany’s extension of its hate speech act. There are also Facebook’s efforts to maintain a political ad archive tool. Each is (partially) a response to concerns of a repeat, in Europe and beyond, of the “fake news” crisis of 2016.

So far, the pandemic and (for some) election-related matters are “serious queries” in the sense that the information returned should not be fully in the hands of current trends in algorithmic culture but returned to editors. With content reviewers and moderators, there is currently a blurring (and in a sense cheapening) of editors, however. Their low-wage, outsourced work to date has had to do with violent and pornographic content rather than the “quality of information” (Roberts, 2019). There is the question of the journalistic training and qualifications for the editing work (Parks, 2019). The professional fact-checking editors, as mentioned above, would struggle with volume.

There are advocates of an editorial recovery online. Source adjudication techniques on offer these days for results curation are, among others, journalistic balance, the absence of biased sources, fact-checked stories, and “longue durée” expertize, be it official and/or established. Crowd-sourcing users to flag inappropriate content or only checking trending content are also available approaches. All mark the return of qualitatively determining the worthiness of source appearance and could be dubbed editorial epistemologies. Each requires particular judgements in advance of the moment of gaveling the A/B or ignore/delete decision, as platforms are wont to decide to allow a post or not. (For world leaders, as mentioned, the posts may be labeled.) There is also the question of handling the volume of posts to be scrutinised.

When curating results or otherwise managing outputs, to undertake “balanced list” work implies making political or partisan source distinctions, and continually returning to the outputs to check the weight of each side per substantive query. An approach seeking an “absence of biased sources” presupposes classification and monitoring and likely relying on official, institutionalized information. Fact-checking, rather than on a source level, switches the efforts to the individual story, and subsequently researches, archives and labels them. At least as it has been performed on Facebook posts by DPA and AFP, the German and French news agencies respectively, it is such meticulous work that it outputs a total of about four fact-checks per day, if their production prior to the 2021 Dutch elections is exemplary (AFP, 2021; DPA, 2021). Relying on “longue durée” expertize could be another means of offering high-quality sources, as organisations working in the same terrain for many years would have accrued credibility, but to official sources it would add non-governmental and other specialized organisations with an established track record (and perhaps a noticeable political leaning).

Another starting point is to take an active audience approach, and assume that another, perhaps more significant instance of filtering lies with the user or what was once known as the “wisdom of the crowd”. Users are able to “flag” or report content on various platforms and label it as inappropriate, misleading, etc. Taking such user reporting practices a step further, as mentioned above, Twitter’s “Birdwatch” program seeks dedicated users (not so unlike Wikipedians, albeit without the non-profit spirit) to sift content and enforce platform rules.

As demonstrated in the empirical research reported above, engagement measures that take into account rating (liking), circulating (sharing) and commenting (reading) are another means to determine the activity of audiences. On Facebook, but also on Twitter (retweeting), one may inquire into the stories about the coronavirus and other issues making audiences active. Adjudicating only those posts with the highest engagement would allow liking and sharing to trigger editorial interest.

Finally, one also could argue for an “anything goes” approach to misinformation, returning to a pre-pandemic algorithmic signals method operated in tandem with standard content moderation, editing out violence, pornography, terrorism and hate. Such a return would appear unlikely as it would imply a regress in content review standards on mainstream platforms. For example, since 2019, Twitter policies cover not *just* violence but its “glorification” (Twitter, 2019), as publicized in a case of the labeling a Donald Trump tweet as such. Indeed, more content types are scrutinized these days. Specifically, since the coronavirus pandemic, the types have been expanded to include “misleading” information.

With respect to identifying such information, Twitter writes, “moving forward, we may use these labels (…) in situations where the risks of harm (…) are less severe but where people may still be confused or misled by the content (Roth and Pickles, 2020). Setting aside for a moment the question of taking social media company utterances at face value (John, 2019), the statement raises the prospect that the new editorial epistemologies, together with the contestation that accompanies their fundaments, may abide beyond the current exceptional information state.

## Data Availability

The original contributions presented in the study are included in the article/supplementary material, further inquiries can be directed to the corresponding author.

## References

[B1] AFP (2021). AFP Factcheck Nederland, Agence France-Presse. Available at: https://factchecknederland.afp.com/list(accessed March 31, 2021).

[B2] Allcott H. Gentzkow M. Yu C. (2019). Trends in the Diffusion of Misinformation on Social media. Res. Polit., 1–8. 10.1177/205316801984855410.3386/w25500

[B3] AllSides (2020). Media Bias Ratings. Available at: https://www.allsides.com/media-bias/media-bias-ratings#ratings (accessed March 31, 2021).

[B4] Barkun M. (2016). Conspiracy Theories as Stigmatized Knowledge. Diogenes 62, 114–120. 10.1177/0392192116669288

[B5] Bartlett J. Krasodomski-Jones A. (2015). Counter-speech: Examining Content that Challenges Extremism Online. Demos. Available at: http://www.demos.co.uk/wp-content/uploads/2015/10/Counter-speech.pdf (accessed March 31, 2021).

[B6] Bengani P. (2019). Hundreds of ‘pink Slime’ Local News Outlets Are Distributing Algorithmic Stories and Conservative Talking Points Tow Center for Journalism, Columbia University. Available at: https://www.cjr.org/tow_center_reports/hundreds-of-pink-slime-local-news-outlets-are-distributing-algorithmic-stories-conservative-talking-points.php (accessed March 31, 2021).

[B7] Benkler Y. Faris R. Roberts H. (2018). Network Propaganda: Manipulation, Disinformation, and Radicalization in American Politics. Oxford: Oxford University Press. 10.1093/oso/9780190923624.001.0001

[B8] Bounegru L. Gray J. Venturini T. Michele M. (2018). A Field Guide to Fake News. Amsterdam: Public Data Lab.

[B9] Bruns A. Harrington S. Hurcombe E. (2020). 'Corona? 5G? or Both?': the Dynamics of COVID-19/5G Conspiracy Theories on Facebook. Media Int. Aust. 177(1). 12–29. 10.1177/1329878X20946113

[B10] Burton A. G. Koehorst D. (2020). Research Note: The Spread of Political Misinformation on Online Subcultural Platforms. HKS Misinfo Rev. 1, 6. 10.37016/mr-2020-40

[B11] BuzzSumo (2020). Buzzsumo media Monitoring. Available at: https://buzzsumo.com (accessed March 31, 2021).

[B12] Callery A. Proulx D. T. (1997). Yahoo! Cataloging the Web. J. Internet Cataloging 1, 57–64. 10.1300/J141v01n01_06

[B13] Coleman K. (2021). Introducing Birdwatch, a Community-Based Approach to Misinformation. Available at; https://blog.twitter.com/en_us/topics/product/2021/introducing-birdwatch-a-community-based-approach-to-misinformation.html (accessed March 31, 2021).

[B14] Colombo G. De Gaetano C. (2020). “5 Dutch Political Instagram,” in The Politics of Social media Manipulation. Editors Rogers R. Niederer S. (Amsterdam: Amsterdam University Press), 147–168. 10.1515/9789048551675-006

[B15] Colombo G. Niederer S. (2021). “The Earnest Platform: US Presidential Campaigns, COVID-19, and Social Issues on Instagram,” in In Mainstreaming the Fringe: How Misinformation Propagates on Social media. Editor Rogers R. (Amsterdam: Amsterdam University Press).

[B16] Cooper Sara. (2020). How to Medical, TikTok Video. Available at: https://www.tiktok.com/@whatchugotforme/video/6819061413877763334 (accessed March 31, 2021).

[B17] Daniels J. (2018). The Algorithmic Rise of the "Alt-Right". Contexts 17, 60–65. 10.1177/1536504218766547

[B18] De Zeeuw D. Tuters M. (2020). Teh Internet Is Serious Business. Cult. Polit. 16, 214–232. 10.1215/17432197-8233406

[B19] Dean J. (1998). Aliens in America. Ithaca, NY: Cornell University Press.

[B20] DiResta R. Shaffer K. Ruppel B. Sullivan D. Matney R. Fox R. (2018). The Tactics & Tropes of the Internet Research Agency. Available at: https://disinformationreport.blob.core.windows.net/disinformation-report/NewKnowledge- Disinformation-Report-Whitepaper.pdf (accessed March 31, 2021). Austin, TX: New Knowledge.

[B21] Donovan J. (2019). How Memes Got Weaponized: A Short History. Available at: https://www.technologyreview.com/2019/10/24/132228/political-war-memes-disinformation/ (accessed March 31, 2021). MIT Technology Review.

[B22] DPA (2021). Dpa Fact-Checking Deutsche Presse-Agentur. Avaialble at: https://dpa-factchecking.com/netherlands/ (accessed March 31, 2021).

[B23] Ellis E. G. (2019). Fighting Instagram’s $1.3 Billion Problem—Fake Followers Wired. 10 September. Available at: https://www.wired.com/story/instagram-fake-followers/ (accessed March 31, 2021).

[B24] Facebook (2018). Coordinated Inauthentic Behavior. Facebook Newsroom. 6 December. Available at: https://about.fb.com/news/2018/12/inside-feed-coordinated-inauthentic-behavior/ (accessed March 31, 2021).

[B25] Facebook (2021). January 2021 Coordinated Inauthentic Behavior Report Facebook Newsroom. 9 Februaryd. Avaialble at: https://about.fb.com/news/2021/02/january-2021-coordinated-inauthentic-behavior-report/ (accessed March 31, 2021).

[B26] Gadde V. Roth Y. (2018). Enabling Further Research of Information Operations on Twitter. Twitter Blog 17 October. Avaialble at: https://blog.twitter.com/en_us/topics/company/2018/enabling-further-research-of-information-operations-on-twitter.html (accessed March 31, 2021).

[B27] Gagliardone I. (2019). Defining Online Hate and its ‘public Lives’: What Is the Place for ‘extreme Speech’? Int. J. Commun. 13, 3068–3087.

[B28] Gallucci N. (2016). 8 Ways to Consume News without Using Facebook. Mashable. 22 November. Available at: https://mashable.com/2016/11/22/consume-news-without-facebook/ (accessed March 31, 2021).

[B29] Gibbs S. (2016). Google Alters Search Autocomplete to Remove ‘are Jews Evil’ Suggestion The Guardian 5 December, Available at: https://www.theguardian.com/technology/2016/dec/05/google-alters-search-autocomplete-remove-are-jews-evil-suggestion (accessed March 31, 2021).

[B30] Gillespie T. (2018). Custodians of the Internet: Platforms, Content Moderation, and the Hidden Decisions that Shape Social media. New Haven: Yale University Press.

[B31] Groen M. Geboers M. (2021). “Fringe Players on Political Twitter: Source-Sharing Dynamics, Partisanship and ‘inauthentic’ Actors before and after the 2020 U.S. Elections, ” in Mainstreaming the Fringe: How Misinformation Propagates on Social media. Editor Rogers R. (Amsterdam: Amsterdam University Press).

[B33] Hedrick A. Karpf D. Kreiss D. (2018). The Earnest Internet vs. The Ambivalent Internet. Available at: https://ijoc.org/index.php/ijoc/article/view/8736 (accessed March 31, 2021). Int. J. Commun. 12, 8.

[B34] Herrman J. (2016). Inside Facebook’s (Totally Insane, Unintentionally Gigantic, Hyperpartisan) Political-media Machine, Available at: https://www.nytimes.com/2016/08/28/magazine/inside-facebooks-totally-insane-unintentionally-gigantic-hyperpartisan-political-media-machine.html (accessed March 31, 2021). 24 August. New York Times.

[B35] Howard P. N. Ganesh B. Liotsiou D. Kelly J. François C. (2018). The IRA, Social media and Political Polarization in the United States, 2012-2018’. Oxford: Computational Propaganda Research Project Oxford Internet Institute.

[B36] HypeAuditor (2020). Instagram Reports. Available at: https://hypeauditor.com/reports/instagram/ (accessed March 31, 2021).

[B37] John N. A. (2019). Social Media Bullshit: What We Don't Know about facebook.Com/peace and Why We Should Care January-March:. Soc. Media + Soc. 5. 1–16. 10.1177/2056305119829863

[B38] Komok A. (2018). How to Check Instagram Account for Fake Followers. Available at: https://hypeauditor.com/blog/how-to-check-instagram-account-for-fake-followers/ (accessed March 31, 2021). HypeAuditor.

[B39] Langville A. N. Meyer C. D. (2006). Google’s PageRank and beyond: The Science of Search Engine Rankings. Princeton: Princeton University Press. 10.1515/9781400830329

[B40] Lewis R. (2018). Alternative Influence: Broadcasting the Reactionary Right on YouTub. Available at: https://datasociety.net/output/alternative-influence/ (accessed March 31, 2021). New York: Data & Society. 10.7591/9781501727672

[B41] Lindquist J. (2018). Illicit Economies of the Internet. Made China J. 3 (4), 88–91. 10.22459/mic.03.04.2018.14

[B42] Mahendran L. Alsherif N. (2020). Adding Clarity to Our Community Guidelines. TikTok Newsroom. 8 January. Available at: https://newsroom.tiktok.com/en-us/adding-clarity-to-our-community-guidelines (accessed March 31, 2021).

[B43] Media Bias/Fact Check (2020). The Most Comprehensive media Bias Resource. Available at: https://mediabiasfactcheck.com (accessed March 31, 2021).

[B44] Mosseri A. (2017). Working to Stop Misinformation and False News. Facebook Newsroom. 6 April. Available at: https://about.fb.com/news/2017/04/working-to-stop-misinformation-and-false-news/ (accessed March 31, 2021).

[B45] NewsGuard (2020). NewsGuard Nutrition Label. NewsGuard. Available at: https://api.newsguardtech.com (accessed March 31, 2021).

[B46] Olmsted K. (2009). Real Enemies: Conspiracy Theories and American Democracy World War I to 9/11. Oxford: Oxford University Press.

[B47] Otero V. (2020). The Chart. Version 3.1. Ad Fontes media. Available at: https://www.adfontesmedia.com/the-chart-version-3-0-what-exactly-are-we-reading/ (accessed March 31, 2021).

[B48] Parks L. (2019). Dirty Data: Content Moderation, Regulatory Outsourcing, and the Cleaners. Film Q. 73, 11–18. 10.1525/fq.2019.73.1.11

[B49] Pepp J. Michaelson E. Sterken R. (2019). Why We Should Keep Talking about Fake News. Inquiry, 1–17. 10.1080/0020174X.2019.1685231

[B50] Phillips W. Milner R. (2017). The Ambivalent Internet: Mischief, Oddity, and Antagonism Online. Cambridge: Polity Press.

[B52] Rheingold H. (1994). The Millennial Whole Earth Catalog. San Francisco: HarperCollins. 10.1037/10146-000

[B53] Roeder O. (2018). We Gave You 3 Million Russian Troll Tweets. Here’s what You’ve Found So Far FiveThirtyEight. 8 August. Available at: https://fivethirtyeight.com/features/what-you-found-in-3-million-russian-troll-tweets/ (accessed March 31, 2021).

[B54] Rogers R. (2020b). Deplatforming: Following Extreme Internet Celebrities to Telegram and Alternative Social media. Eur. J. Commun. 35, 213–229. 10.1177/0267323120922066

[B55] Rogers R. (2013). Digital Methods. Cambridge, MA: MIT Press. 10.7551/mitpress/8718.001.0001

[B56] Rogers R. Hagen S. (2020). “9 Epilogue,” in The Politics of Social media Manipulation. Editors Rogers R. Niederer S. (Amsterdam: Amsterdam University Press), 253–256. 10.1515/9789048551675-010

[B57] Rogers R. (2004). Information Politics on the Web. Cambridge, MA: MIT Press. 10.7551/mitpress/3770.001.0001

[B58] Rogers R. (2021). Mainstreaming the Fringe: How Misinformation Propagates on Social media. Amsterdam: Amsterdam University Press.

[B59] Rogers R. (2020a). Research Note: The Scale of Facebook's Problem Depends upon How 'fake News' Is Classified. HKS Misinfo Rev. 1, 6. 10.37016/mr-2020-43

[B60] Romm T. Stanley-Becker I. (2019). Facebook, Twitter Disable Sprawling Inauthentic Operation that Used AI to Make Fake Faces Washington Post. 21 December. Available at: https://www.washingtonpost.com/technology/2019/12/20/facebook-twitter-disable-sprawling-inauthentic-operation-that-used-ai-make-fake-faces/ (accessed March 31, 2021).

[B61] Roth Y. Pickles N. (2020). Updating Our Approach to Misleading Information. Twitter Blog 11 May. Available at: https://blog.twitter.com/en_us/topics/product/2020/updating-our-approach-to-misleading-information.html (accessed March 31, 2021).

[B62] Sánchez-Querubín N. Wang S. Dickey B. Benedetti A. (2021). “TikTok: Remix, Drama, and Misinformation,” in In Mainstreaming the Fringe: How Misinformation Propagates on Social media. Editor Rogers R. (Amsterdam: Amsterdam University Press).

[B63] Schwartz O. (2018). Are Google and Facebook Really Suppressing Conservative Politics? the Guardian 4 December. Available at: https://www.theguardian.com/technology/2018/dec/04/google-facebook-anti-conservative-bias-claims (accessed March 31, 2021).

[B64] Shirky C. (2008). Here Comes Everybody. New York: Penguin.

[B65] Silverman C. Alexander L. (2016). How Teens in the Balkans Are Duping Trump Supporters with Fake News. Buzzfeed News 3 November. Available at: https://www.buzzfeednews.com/article/craigsilverman/how-macedonia-became-a-global-hub-for-pro-trump-misinfo (accessed.March 31, 2021).

[B66] Silverman C. (2016). This Analysis Shows How Viral Fake Election News Stories Outperformed Real News on Facebook. Buzzfeed News. 16 November. Available at: https://www.buzzfeednews.com/article/craigsilverman/viral-fake-election-news-outperformed-real-news-on-facebook (accessed March 31, 2021).

[B67] Sparktoro (2021). Audience Intelligence. Available at: https://sparktoro.com (accessed March 31, 2021).

[B68] Sullivan D. (2004). Google in Controversy over Top-Tanking for Anti-jewish Site Search Engine Watch. 24 April. Available at: https://www.searchenginewatch.com/2004/04/24/google-in-controversy-over-top-ranking-for-anti-jewish-site/ (accessed March 31, 2021).

[B69] Tarkov A. (2012). Journatic Worker Takes ‘This American Life’ inside Outsourced Journalism. Poynter. 30 June. Available at: https://www.poynter.org/reporting-editing/2012/journatic-staffer-takes-this-american-life-inside-outsourced-journalism/ (accessed March 31, 2021).

[B70] Torres G. (2021). “Problematic Information in Google Web Search? Scrutinizing the Results from U.S. Election-Related Queries,” in Mainstreaming the Fringe: How Misinformation Propagates on Social media. Editor Rogers R. (Amsterdam: Amsterdam University Press).

[B71] Tuters M. Burton A. (2021). The Rebel Yell: Toxic Vox Populism on YouTube. forthcoming: Canadian Journal of Communication.

[B72] Twitter (2019). ‘Glorification of Violence Policy’, Twitter Help center. Available at: https://help.twitter.com/en/rules-and-policies/glorification-of-violence.

[B73] Tynan D. (2016). How Facebook powers Money Machines for Obscure Political ‘news’ Sites, August. The Guardian. Available at: https://www.theguardian.com/technology/2016/aug/24/facebook-clickbait-political-news-sites-us-election-trump (accessed March 31, 2021).24.

[B74] UN DGC (2020). UN Tackles ‘infodemic’ of Misinformation and Cybercrime in COVID-19 Crisis. UN Department of Global Communications, 31. March. Available at: https://www.un.org/en/un-coronavirus-communications-team/un-tackling-’infodemic’-misinformation-and-cybercrime-covid-19 (accessed March 31, 2021).

[B75] Vaidhyanathan S. (2019). Why Conservatives Allege Big Tech Is Muzzling Them The Atlantic 28 July. Available at: https://www.theatlantic.com/ideas/archive/2019/07/conservatives-pretend-big-tech-biased-against-them/594916/ (accessed March 31, 2021).

[B76] Van der Linden S. Panagopoulos C. Roozenbeek J. (2020). You Are Fake News: Political Bias in Perceptions of Fake News. Media, Cult. Soc, 42. 460–470. 10.1177/0163443720906992

[B77] Venturini T. (2019). “From Fake to Junk News,” in In Data Politics: Worlds, Subjects, Rights. Editors Bigo D. Isin E. Ruppert E. (London: Routledge)), 123–144. 10.4324/9781315167305-7

[B78] Wardle C. (2017). Fake News: It’s Complicated. First Draft. 16 February. Available at: https://firstdraftnews.org/latest/fake-news-complicated/ (accessed March 31, 2021).

[B79] Wikipedia contributors (2020). Killian Documents Controversy. Wikipedia: The Free Encyclopaedia. Available at: https://en.wikipedia.org/w/index.php?title=Killian_documents_authenticity_issues&oldid=962589844 (accessed March 31, 2021).

[B80] Wilkinson W. W. Berry S. D. (2020). Together They Are Troy and Chase: Who Supports Demonetization of Gay Content on YouTube? Psychol. Popular Media 9, 224–235. 10.1037/ppm0000228

